# Embodied Cognition: A Challenging Road for Clinical Neuropsychology

**DOI:** 10.3389/fnagi.2017.00388

**Published:** 2017-11-22

**Authors:** Juan F. Cardona

**Affiliations:** Instituto de Psicología, Universidad del Valle, Santiago de Cali, Colombia

**Keywords:** embodied cognition, clinical neuropsychology, neuropsychological tests, modularity of mind, cognitive assessment

Clinical neuropsychology (CN) has clearly emerged as a diagnostic discipline, influenced by *disembodied and localizationist-connectionist approaches*. In this sense, cognition has been understood as a relatively isolated link of perception and movement. The classic model “perception → cognition → action” has guided the design of instruments and the interpretation of the results in pathological conditions of the central nervous system. This trend has been maintained over time thanks to the convergence of the localizationist approach and computational models of information processing adopted by CN (Shallice, 1988).

Recently, embodied cognition (EC) has put the sensory-motor system on the stage of human cognitive neuroscience (Willems and Francken, 2012; Freund et al., 2016). EC proposes that the brain systems underlying perception and action are integrated with cognition in bidirectional pathways (Borghi and Pecher, 2011; Ibáñez et al., 2013; Cardona et al., 2014), highlighting their connection with bodily (Gallese and Lakoff, 2005; Gallese and Sinigaglia, 2011) and emotional (Niedenthal, 2007; De Jaegher et al., 2010) experiences, leading to research programs aimed at demonstrating the influence of action on perception (Proffitt, 2006; Creem-Regehr and Kunz, 2010; Witt, 2011) and high-level cognition (Barrett et al., 2007; Goldin-Meadow and Beilock, 2010; Ibáñez and Manes, 2012).

Embodied cognition (EC) findings are gradually leading to an integrative view of mental functions and their interdependence with the context, questioning the validity of the computational paradigm and the localizationalist doctrine in explaining brain functioning and its correspondence with complex behavior. These findings have also given a scientific character to the study of bodily experience in the analysis of brain function. The continuous accumulation of evidence from EC has had both theoretical and experimental influence and is expected to ultimately impact the clinical field. This article presents some of the main challenges that neuropsychology faces in integrating EC in its clinical assessment and diagnosis processes.

## Cerebral boundaries: roots of neuropsychological assessment

Historically, perception and movement have been separated from cognition. The events that occurred in the nineteenth century are a turning point in the parsed study of the functions of the nervous system. The neurophysiology of the motor and sensory processes was consolidated from the pioneering findings of Bell (1811) and Magendie (1822). With the same importance in CN, Dax (1836) (See, Cubelli and Montagna, 1994; Manning and Thomas-Anterion, 2011) and Broca (1861) reported the correspondence between alterations in language production and damage to the left frontal zone of the brain, consolidating the localizationist approach in the explanation of cognitive organization.

Although alternative proposals such as the equipotential theory (Lashley, 1929) that emphasized the interactivity of the whole brain (holism) emerged, localizationalist doctrine and its methods were extended at an accelerated rate to the study of various cognitive functions, making it impossible to trace a single line of scientific development from that period.

In this way, the diagnostic procedures and methods of investigation in CN were directly influenced by localizationist theory. Pioneering neuropsychologists, including Henry Hecaen, Oliver Zangwill, Hans-Lukas Teuber and Brenda Milner, worked together with neurosurgeons to develop reliable tools that would discriminate patients with brain lesions from normal controls (Halstead, 1947; Hartlage, 1966). The approach between behavioral neurology and psychometrics led to the selection of sensitive tests to find anatomic-clinical correspondences contained in classical neuropsychological batteries, such as the Halstead-Reitan (Halstead, 1947; Reitan and Wolfson, 1985, 1993) Luria-Nebraska (Golden et al., 1980) and other instruments used in clinical practice (Strauss et al., 2006).

A methodology associated with strict localizationism has been the double dissociation (Teuber, 1955; Weiskrantz, 1968, 1991), which is shown when lesions in area A affect function X more than function Y, while lesions in area B affect function Y more than function X. This type of approach responds to a logic of “effect = structure” and suggests that performance in a given task depends on a specialized area of the cerebral cortex (Pribram, 1971).

The convergence between the modular theory of Fodor (1983) and the advent of neuroimaging techniques gave credence to this fragmented brain organization, leading to the overvaluation of strong dissociations between brain functions such as hearing (Peretz et al., 1994; Peretz and Coltheart, 2003), memory (Moscovitch, 1995; Moscovitch and Nachson, 1995) and language (Warrington and Shallice, 1984; Warrington and McCarthy, 1987; Pinker and Ullman, 2002; Crutch and Warrington, 2003) and to the selection of numerous tasks aimed at inferring the delimited location of brain damage that still have clinical utility in the recognition of cognitive alterations (Strauss et al., 2006; Lezak, 2012). Although the development of measuring instruments represented an advance in the CN, many tasks cannot easily be extrapolated to activities of daily living. To overcome this problem, important advances have been made in the development of tests that have greater ecological validity, establishing a better relationship between the evaluation and the interpretation of the results, taking into account the impact that cognitive alterations have in the everyday activities of patients (Wilson et al., 1985, 1987; McDonald et al., 2003; Torralva et al., 2009). However, perception and action are still isolated from cognitive domains.

## An integrative view of brain-context interaction

The idea of coupling between mental processes is not new in CN. In fact, clinical evidence from the pioneering work of Gonzalo (1950) (see Gonzalo-Fonrodona, 2009) and Goldberg (1989, 1995) suggested a dynamic organization of the cerebral cortex underlying sensory, cognitive and motor integration. These findings have been supported by cross-modal studies (Fuster et al., 2000; Ghazanfar and Schroeder, 2006; Driver and Noesselt, 2008; Spence and Parise, 2012) that show interactivity and coupling in processes such as vision (Klapetek et al., 2012), hearing (Parise and Spence, 2012), somato-perception and the chemical senses (Hanson-Vaux et al., 2013).

However, it was the discovery of mirror neuron system (MNS) in the human brain (Rizzolatti and Craighero, 2004) that facilitated EC to show the interaction of language and motor system (Rizzolatti and Arbib, 1998; Pulvermuller, 2005; Arévalo et al., 2015). In light of these findings, neuropsychological exploration in diseases traditionally classified as movement disorders has uncovered a compromise in action language processing (Bak, 2013), such as corticobasal degeneration (Cotelli et al., 2006; Silveri and Ciccarelli, 2007), Huntington's disease (Peran et al., 2004; Kargieman et al., 2014), progressive supranuclear palsy (Bak et al., 2006; Cotelli et al., 2006), amyotrophic lateral sclerosis (Neary et al., 2000; Bak et al., 2001; Bak and Hodges, 2004) and Parkinson's disease (Boulenger et al., 2008; Cardona et al., 2013, 2014), revealing the compromise of language even at an early stage of pathology and becoming a potential early marker in the specific case of Parkinson's disease.

Additionally, under the EC framework, the study of emotional processing, understood as a multilevel and context-dependent domain (Barrett et al., 2007), has been given a prominent place, making it clear how neurodegenerative pathologies (Ibáñez and Manes, 2012) and neuropsychiatric disorders (Ibáñez et al., 2011) present notorious difficulties when integrating contextual cues into ecological cognitive tasks.

## Conclusion

Embodied cognition (EC) findings in the area of neuropsychology demonstrate that interactivity and multimodality are fundamental features of brain functioning and that contextual interdependence can be a crucial factor in regard to bringing about effective interventions, opening the door to new research in various clinical conditions. This premise reflects a methodological imperative for CN.

First, the model of cerebral functioning that guides neuropsychological intervention should be strengthened in consensus. In this sense, EC invites an integrative vision of mental processes and encourages the development of new methods that complement the approaches of cognitive functions. Although current assessment tools are sensitive and reliable in detecting cognitive deficits associated with central nervous system disorders, the design and integration of multimodal, emotional and contextual processing measures may have more ecological validity than other measures (Franzen and Wilhelm, 1996; Chaytor and Schmitter-Edgecombe, 2003; Rabin et al., 2007).

Second, the findings of linkages between the language and motor systems could guide the establishment of clinical guidelines that include the evaluation of the language of action in neurodegenerative motor pathologies and could also provide new perspectives and intervention strategies that may delay cognitive impairment in these clinical pictures. This suggestion is based on the positive results of cognitive stimulation in reducing executive and functional deficits in patients with Parkinson's disease (Sinforiani et al., 2004; Sammer et al., 2006). In this way, it is necessary to clarify the different deficits in language processing to propose cross-modal treatments that facilitate the attenuation of motor deficits. Likewise, the establishment of the relationship between the language and motor systems leads to the need to verify the presence of other couplings between the sensory-motor system and other domains of cognition to enable the establishment of rehabilitation programs.

Embodied cognition (EC) also provides new perspectives and intervention strategies in other areas of mental health. Some proposals have emerged in clinical psychology (Zatti and Zarbo, 2015) and psychiatry (Fuchs, 2009; Fuchs and Schlimme, 2009) in which classical theories of cognition presented serious limitations in explaining the appearance of non-underlying behavioral alterations to specific brain injuries and in which experience and environmental factors play fundamental roles.

The integration of experience and context into the triad “perception, cognition and action” has been previously accepted in the understanding of brain development in childhood. However, EC findings show that the adult brain remains context-dependent and that experience directly impacts both structural and brain functioning throughout life. Although localizational and computational models have made numerous contributions to the development of clinical practice, EC highlights the role of the body and the sensory-motor experience, allowing a broader vision and a better understanding of cognitive processes under both normal and pathological conditions.

## Author contributions

The author confirms being the sole contributor of this work and approved it for publication.

### Conflict of interest statement

The author declares that the research was conducted in the absence of any commercial or financial relationships that could be construed as a potential conflict of interest.

## References

[B1] Arévalo A. Baldo J. González-Perilli F. Ibáñez A. (2015). Editorial: What can we make of theories of embodiment and the role of the human mirror neuron system? Front. Hum. Neurosci. 9:500. 10.3389/fnhum.2015.00500 26441598 PMC4565976

[B2] Bak T. H. (2013). The neuroscience of action semantics in neurodegenerative brain diseases. Curr. Opin. Neurol. 26, 671–677. 10.1097/WCO.0000000000000039 24184973

[B3] Bak T. H. Hodges J. R. (2004). The effects of motor neurone disease on language: further evidence. Brain Lang. 89, 354–361. 10.1016/S0093-934X(03)00357-2 15068918

[B4] Bak T. H. O'Donovan D. G. Xuereb J. H. Boniface S. Hodges J. R. (2001). Selective impairment of verb processing associated with pathological changes in Brodmann areas 44 and 45 in the motor neurone disease-dementia-aphasia syndrome. Brain 124. 103–120. 10.1093/brain/124.1.103 11133791

[B5] Bak T. H. Yancopoulou D. Nestor P. J. Xuereb J. H. Spillantini M. G. Pulvermüller F.. (2006). Clinical, imaging and pathological correlates of a hereditary deficit in verb and action processing. Brain 129. 321–332. 10.1093/brain/awh701 16330501

[B6] Barrett L. F. Lindquist K. A. Gendron M. (2007). Language as context for the perception of emotion. Trends Cogn Sci. 11, 327–332. 10.1016/j.tics.2007.06.003 17625952 PMC2225544

[B7] Bell C. (1811). Idea of a New Anatomy of the Brain Submitted for the Observation of his Friends. London: Strahan and Preston.

[B8] Borghi A. M. Pecher D. (2011). Introduction to the special topic embodied and grounded cognition. Front. Psychol. 2:187 10.3389/fpsyg.2011.00187 21887149 PMC3156979

[B9] Boulenger V. Mechtouff L. Thobois S. Broussolle E. Jeannerod M. Nazir T. A. (2008). Word processing in Parkinson's disease is impaired for action verbs but not for concrete nouns. Neuropsychologia 46, 743–756. 10.1016/j.neuropsychologia.2007.10.007 18037143

[B10] Broca P. (1861). Remarques sur le siege de la faculte du langage articule; suivies d'une observation d'aphemie. Bull. Soc. Anatom. Paris 6, 330–357.

[B11] Cardona J. F. Gershanik O. Gelormini-Lezama C. Houck A. L. Cardona S. Kargieman L.. (2013). Action-verb processing in Parkinson's disease: new pathways for motor-language coupling. Brain Struct. Funct. 218, 1355–1373. 10.1007/s00429-013-0510-1 23412746

[B12] Cardona J. F. Kargieman L. Sinay V. Gershanik O. Gelormini C. Amoruso L.. (2014). How embodied is action language? Neurological evidence from motor diseases. Cognition 131, 311–322. 10.1016/j.cognition.2014.02.001 24594627

[B13] Chaytor N. Schmitter-Edgecombe M. (2003). The ecological validity of neuropsychological tests: a review of the literature on everyday cognitive skills. Neuropsychol. Rev. 13, 181–197. 10.1023/B:NERV.0000009483.91468.fb 15000225

[B14] Cotelli M. Borroni B. Manenti R. Alberici A. Calabria M. Agosti C.. (2006). Action and object naming in frontotemporal dementia, progressive supranuclear palsy, and corticobasal degeneration. Neuropsychology 20, 558–565. 10.1037/0894-4105.20.5.558 16938018

[B15] Creem-Regehr S. H. Kunz B. R. (2010). Perception and action. Wiley Interdiscip. Rev. Cogn. Sci. 1, 800–810. 10.1002/wcs.82 26271778

[B16] Crutch S. J. Warrington E. K. (2003). The selective impairment of fruit and vegetable knowledge:amultiple processing channels account of fine-grain category specificity. Cogn. Neuropsychol. 20, 355–372. 10.1080/02643290244000220 20957575

[B17] Cubelli R. Montagna C. G. (1994). A reappraisal of the controversy of Dax and Broca. J. Hist. Neurosci. 3, 215–226. 10.1080/09647049409525614 11618822

[B18] Dax M. (1836). Lésions de la moitié gauche de l'encéphale coïncidant avec l'oubli des signes de la pensée (lu á Montpellier en 1836). Bull. Hebd. Med. Chir. 2, 259–262.

[B19] De Jaegher H. Di Paolo E. Gallagher S. (2010). Can social interaction constitute social cognition? Trends Cogn. Sci. 14, 441–447. 10.1016/j.tics.2010.06.009 20674467

[B20] Driver J. Noesselt T. (2008). Multisensory interplay reveals crossmodal influences on ‘sensory-specific’ brain regions, neural responses, and judgments. Neuron 57, 11–23. 10.1016/j.neuron.2007.12.013 18184561 PMC2427054

[B21] Fodor J. A. (1983). The modularity of mind. Cambridge, MA: MIT Press.

[B22] Franzen M. D. Wilhelm K. L. (1996). Conceptual foundations of ecological validity in neuropsychological assessment, in Ecological Validity of Neuropsychological Testing, eds Sbordone R. J. Long C. J. (Boca Raton, FL: St. Lucie Press), 91–112.

[B23] Freund P. Friston K. Thompson A. J. Stephan K. E. Ashburner J. Bach D. R.. (2016). Embodied neurology: an integrative framework for neurological disorders. Brain 139, 1855–1861. 10.1093/brain/aww076 27105896 PMC4892755

[B24] Fuchs T. (2009). Embodied cognitive neuroscience and its consequences for psychiatry. Poiesis Prax. 6, 219–233. 10.1007/s10202-008-0068-9

[B25] Fuchs T. Schlimme J. E. (2009). Embodiment and psychopathology: a phenomenological perspective. Curr. Opin. Psychiatry 22, 570–575. 10.1097/YCO.0b013e3283318e5c 19730373

[B26] Fuster J. M. Bodner M. Kroger J. K. (2000). Cross-modal and cross-temporal association in neurons of frontal cortex. Nature 405, 347–351. 10.1038/35012613 10830963

[B27] Gallese V. Lakoff G. (2005). The Brain's concepts: the role of the Sensory-motor system in conceptual knowledge. Cogn. Neuropsychol. 22, 455–479. 10.1080/02643290442000310 21038261

[B28] Gallese V. Sinigaglia C. (2011). What is so special about embodied simulation? Trends Cogn Sci. 15, 512–519. 10.1016/j.tics.2011.09.003 21983148

[B29] Ghazanfar A. A. Schroeder C. E. (2006). Is neocortex essentially multisensory? Trends Cogn Sci. 10, 278–285. 10.1016/j.tics.2006.04.008 16713325

[B30] Goldberg E. (1989). Gradiental approach to neocortical functional organization. J. Clin. Exp. Neuropsychol. 11, 489–517. 10.1080/01688638908400909 2474566

[B31] Goldberg E. (1995). Rise and fall of modular orthodoxy. J. Clin. Exp. Neuropsychol. 17, 193–208. 10.1080/01688639508405118 7629267

[B32] Golden C. J. Hammeke T. A. Purisch A. D. (1980). The Luria-Nebraska battery manual. Los Angeles, CA: Western Psychological Services.

[B33] Goldin-Meadow S. Beilock S. L. (2010). Action's influence on thought: the case of gesture. Perspect. Psychol. Sci. 5, 664–674. 10.1177/1745691610388764 21572548 PMC3093190

[B34] Gonzalo J. (1950). Investigaciones Sobre la Nueva Dinámica Cerebral. La Actividad Cerebral en Función de las Condiciones Dinámicas de la Excitabilidad Nerviosa. Madrid: Consejo Superior de Investigaciones Científicas.

[B35] Gonzalo-Fonrodona I. (2009). Functional gradients through the cortex, multisensory integration and scaling laws in brain dynamics. Neurocomputing 72, 831–838. 10.1016/j.neucom.2008.04.055

[B36] Halstead W. C. (1947). Brain and Intelligence; A Quantitative Study of the Frontal Lobes. Chicago, IL: University of Chicago Press.

[B37] Hanson-Vaux G. Crisinel A. S. Spence C. (2013). Smelling shapes: crossmodal correspondences between odors and shapes. Chem. Senses 38, 161–166. 10.1093/chemse/bjs087 23118203

[B38] Hartlage L. (1966). Common psychological tests applied to the assessment of brain damage. J. Proj. Tech. Pers. Assess. 30, 317–338. 10.1080/0091651X.1966.10120322 5338863

[B39] Ibáñez A. Cardona J. F. Dos Santos Y. V. Blenkmann A. Aravena P. Roca M.. (2013). Motor-language coupling: direct evidence from early Parkinson's disease and intracranial cortical recordings. Cortex 49, 968–984. 10.1016/j.cortex.2012.02.014 22482695

[B40] Ibáñez A. Manes F. (2012). Contextual social cognition and the behavioral variant of frontotemporal dementia. Neurology 78, 1354–1362. 10.1212/WNL.0b013e3182518375 22529204 PMC3335455

[B41] Ibáñez A. Riveros R. Aravena P. Vergara V. Cardona J. F. García L.. (2011). When context is difficult to integrate: cortical measures of congruency in schizophrenics and healthy relatives from multiplex families. Schizophr Res. 126, 303–305. 10.1016/j.schres.2010.04.008 20472401

[B42] Kargieman L. Herrera E. Baez S. García A. M. Dottori M. Gelormini C.. (2014). Motor–language coupling in Huntington's disease families. Front. Aging Neurosci. 6:122. 10.3389/fnagi.2014.00122 24971062 PMC4054328

[B43] Klapetek A. Ngo M. K. Spence C. (2012). Does crossmodal correspondence modulate the facilitatory effect of auditory cues on visual search? Atten. Percept. Psychophys. 74, 1154–1167. 10.3758/s13414-012-0317-9 22648604

[B44] Lashley K. S. (1929). Brain Mechanism and Intelligence. Chicago, IL: University of Chicago Press.

[B45] Lezak M. D. (2012). Neuropsychological Assessment, 5 Edn, New York, NY: Oxford University Press.

[B46] Magendie F. (1822). Expériences sur les fonctions des racines des nerfs qui naissent de la moelle épinière. J. Physiol. Exp. Pathol. 2, 366–371.

[B47] Manning L. Thomas-Anterion C. (2011). Marc Dax and the discovery of the lateralisation of language in the left cerebral hemisphere. Rev. Neurol. 167, 868–872. 10.1016/j.neurol.2010.10.017 21640366

[B48] McDonald S. Flanagan S. Rollins J. Kinch J. (2003). TASIT: a new clinical tool for assessing social perception after traumatic brain injury. J. Head Trauma Rehabil. 18, 219–238. 10.1097/00001199-200305000-00001 12802165

[B49] Moscovitch M. (1995). Recovered consciousness: a hypothesis concerning modularity and episodic memory. J. Clin. Exp. Neuropsychol. 17, 276–290. 10.1080/01688639508405123 7629272

[B50] Moscovitch M. Nachson I. (1995). Modularity and the brain. Intro. J. Clin. Exp. Neuropsychol. 17, 167–170. 10.1080/01688639508405116 7629265

[B51] Neary D. Snowden J. S. Mann D. M. (2000). Cognitive change in motor neurone disease/amyotrophic lateral sclerosis (MND/ALS). J. Neurol. Sci. 180, 15–20. 10.1016/S0022-510X(00)00425-1 11090859

[B52] Niedenthal P. M. (2007). Embodying emotion. Science 316, 1002–1005. 10.1126/science.1136930 17510358

[B53] Parise C. V. Spence C. (2012). Audiovisual crossmodal correspondences and sound symbolism: a study using the implicit association test. Exp. Brain Res. 220, 319–333. 10.1007/s00221-012-3140-6 22706551

[B54] Péran P. Démonet J. F. Pernet C. Cardebat D. (2004). Verb and noun generation tasks in Huntington's disease. Mov. Disord. 19, 565–571. 10.1002/mds.10706 15133822

[B55] Peretz I. Coltheart M. (2003). Modularity of music processing. Nat. Neurosci. 6, 688–691. 10.1038/nn1083 12830160

[B56] Peretz I. Kolinsky R. Tramo M. Labrecque R. Hublet C. Demeurisse G.. (1994). Functional dissociations following bilateral lesions of auditory cortex. Brain 117, 1283–1301. 10.1093/brain/117.6.1283 7820566

[B57] Pinker S. Ullman M. T. (2002). The past and future of the past tense. Trends Cogn. Sci. 6, 456–463. 10.1016/S1364-6613(02)01990-3 12457895

[B58] Pribram K. H. (1971). Languages of the Brain. Upper Saddle River, NJ: Prentice-Hall.

[B59] Proffitt D. R. (2006). Embodied perception and the economy of action. Perspect. Psychol. Sci. 1, 110–122. 10.1111/j.1745-6916.2006.00008.x 26151466

[B60] Pulvermüller F. (2005). Brain mechanisms linking language and action. Nat. Rev. Neurosci. 6, 576–582. 10.1038/nrn1706 15959465

[B61] Rabin L. A. Burton L. A. Barr W. B. (2007). Utilization rates of ecologically oriented instruments among clinical neuropsychologists. Clin. Neuropsychol. 21, 727–743. 10.1080/13854040600888776 17676540

[B62] Reitan R. M. Wolfson D. (1985). Halstead-Reitan Neuropsychological test battery: Theory and Clinical Interpretation. Tucson, AZ: Neuropsychology Press.

[B63] Reitan R. M. Wolfson D. (1993). The Hasltead-Reitan Neuropsychological Test Battery: Theory and Clinical Interpretation.. Tucson, AZ: Neuropsychology Press.

[B64] Rizzolatti G. Arbib M. A. (1998). Language within our grasp. Trends Neurosci. 21, 188–194. 10.1016/S0166-2236(98)01260-0 9610880

[B65] Rizzolatti G. Craighero L. (2004). The mirror-neuron system. Annu. Rev. Neurosci. 27, 169–192. 10.1146/annurev.neuro.27.070203.144230 15217330

[B66] Sammer G. Reuter I. Hullmann K. Kaps M. Vaitl D. (2006). Training of executive functions in Parkinson's disease. J. Neurol. Sci. 248, 115–119. 10.1016/j.jns.2006.05.028 16765378

[B67] Shallice T. (1988). From Neuropsychology to Mental Structure. New York, NY: Cambridge University Press.

[B68] Silveri M. C. Ciccarelli N. (2007). The deficit for the word-class “verb” in corticobasal degeneration: linguistic expression of the movement disorder? Neuropsychologia 45, 2570–2579. 10.1016/j.neuropsychologia.2007.03.014 17467749

[B69] Sinforiani E. Banchieri L. Zucchella C. Pacchetti C. Sandrini G. (2004). Cognitive rehabilitation in Parkinson's disease. Arch Gerontol. Geriatr. 9(Suppl.1), 387–391. 10.1016/j.archger.2004.04.049 15207437

[B70] Spence C. Parise C. V. (2012). The cognitive neuroscience of crossmodal correspondences. Iperception 3, 410–412. 10.1068/i0540ic 23145291 PMC3485837

[B71] Strauss E. Sherman E. M. Spreen O. (2006). A Compendium of Neuropsychological Tests: Administration, Norms and Commentary, 3rd Edn., New York, NY: Oxford University Press.

[B72] Teuber H. L. (1955). Physiological psychology. Annu. Rev. Psychol. 6, 267–296. 10.1146/annurev.ps.06.020155.001411 14377367

[B73] Torralva T. Roca M. Gleichgerrcht E. Bekinschtein T. Manes F. (2009). A neuropsychological battery to detect specific executive and social cognitive impairments in early frontotemporal dementia. Brain 132, 1299–1309. 10.1093/brain/awp041 19336463

[B74] Warrington E. K. McCarthy R. A. (1987). Categories of knowledge. Further fractionations and an attempted integration. Brain 110, 1273–1296. 10.1093/brain/110.5.1273 3676701

[B75] Warrington E. K. Shallice T. (1984). Category specific semantic impairments. Brain 107, 829–854. 10.1093/brain/107.3.829 6206910

[B76] Weiskrantz L. (1968). Treatments, inference and brain function, in Analysis of Behavioural Change, ed Weiskrantz L. (New York, NY: Harper and Row), 400–414.

[B77] Weiskrantz L. (1991). Dissociations and associates in neuropsychology, in Perspectives in Cognitive Neuroscience, ed Lister R. G. (New York, NY: Oxford University Press), 847–864.

[B78] Willems R. M. Francken J. C. (2012). Embodied cognition: taking the next step. Front. Psychol. 3:582. 10.3389/fpsyg.2012.00582 23293620 PMC3531608

[B79] Wilson B. A. Cockburn J. Baddeley A. (1985). The Rivermead Behavioral Memory Test. Reading, England: Thames Valley Test Co. Gaylord, MI: National Rehabilitation Services.

[B80] Wilson B. A. Cockburn J. Halligan P. (1987). Behavioural Inattention Test. Titchfield, Fareham, Hants, England: Thames Valley Test Co. Gaylord, MI: National Rehabilitation Services.

[B81] Witt J. K. (2011). Action's effects on perception. Curr. Dir. Psychol. Sci. 20, 201–206. 10.1177/0963721411408770

[B82] Zatti A. Zarbo C. (2015). Embodied and exbodied mind in clinical psychology. A proposal for a psycho-social interpretation of mental disorders. Front. Psychol. 6:236. 10.3389/fpsyg.2015.00236 25784894 PMC4347298

